# Supplementary material for: Regulatory B Cells Involvement in Autoimmune Phenomena Occurring in Pediatric Graves’ Disease Patients
Source: Int J Mol Sci. 2021 Oct 10;22(20):10926. doi: 10.3390/ijms222010926 (PMC8536076; doi:10.3390/ijms222010926)
Supplement: Supplementary file 1 [file ijms-22-10926-s001.zip › Supplementary Fig S2.pdf]

a

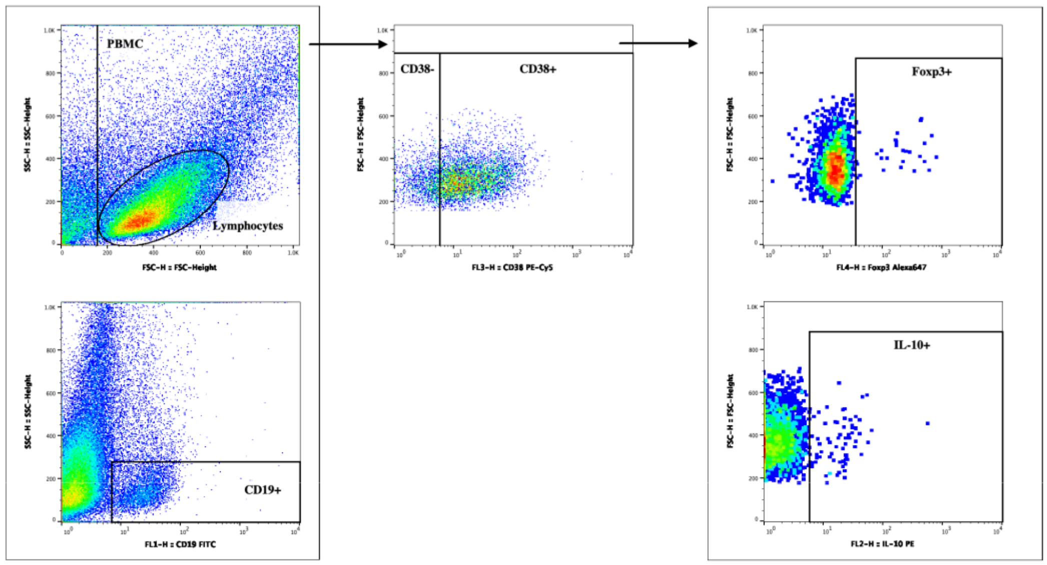

b

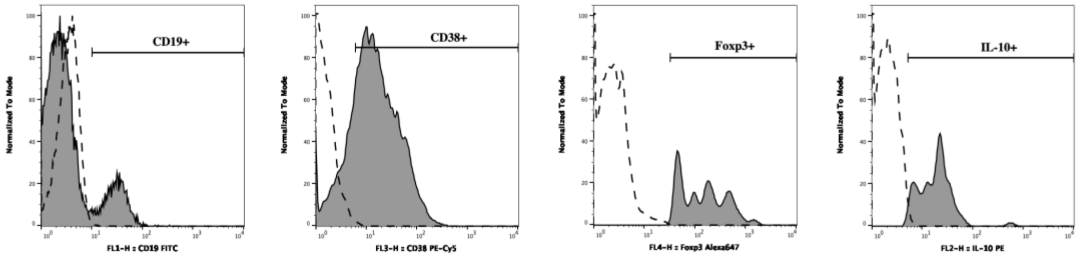

c

**Antibody**	**CD19**	**IL-10**	**CD38**	**Foxp3**
**Reactivity**	Human	Human	Human	Human
**Clone**	HIB19	JES3-9D7	HIT2	259D/C7
**Host**	Mouse	Rat	Mouse	Mouse
**Fluorochrome**	FITC	PE	PE-Cy5	Alexa Fluor 647

Supp. Fig S2. Representative gating strategy implemented in Bregs assessment in Graves patients. Regulatory B cells were distinguished on the basis of morphological properties (FSC/SSC) and presence of surface CD19, followed by gating of CD38-/CD38+ cells and analysis of Foxp3 and/or IL-10 within each population (a). Gating for all the markers used in the study was based on the proper FMO and unstained controls (b). Detailed specification of the monoclonal antibodies used was presented in the table (c).
