# Supplementary material for: Regulatory B Cells Involvement in Autoimmune Phenomena Occurring in Pediatric Graves’ Disease Patients
Source: Int J Mol Sci. 2021 Oct 10;22(20):10926. doi: 10.3390/ijms222010926 (PMC8536076; doi:10.3390/ijms222010926)
Supplement: Supplementary file 1 [file ijms-22-10926-s001.zip › Supplementary Tab. S1.pdf]

a

	Graves-Basedow (GB) Time 0	Graves-Basedow (GB) Time 1	Graves-Basedow (GB) Time 2	Control group (HC)	Normal range
Age [years]	13.1 (± 4.011)			12.42 (± 3.457)	
Sex distribution [male to female]	18% to 82%			40% to 60%	
TSH [mIU/l]	0.598 (± 1.273)	0.818 (± 1.064)	6.706 (± 11.54)	2.245 (± 1.128)	0.32 – 5.0
fT4 [ng/dl]	4.655 (± 3.146)	1.185 (± 0.677)	1.037 (± 0.595)		0.71 – 1.55
fT3 [ng/l]	19.25 (± 12.77)	3.855 (± 1.637)	3.880 (± 2.222)		2.60 – 5.40
TRAb [IU/l]	25.93 (± 13.16)	13.93 (± 12.26)	7.743 (± 5.643)		< 1.7

b

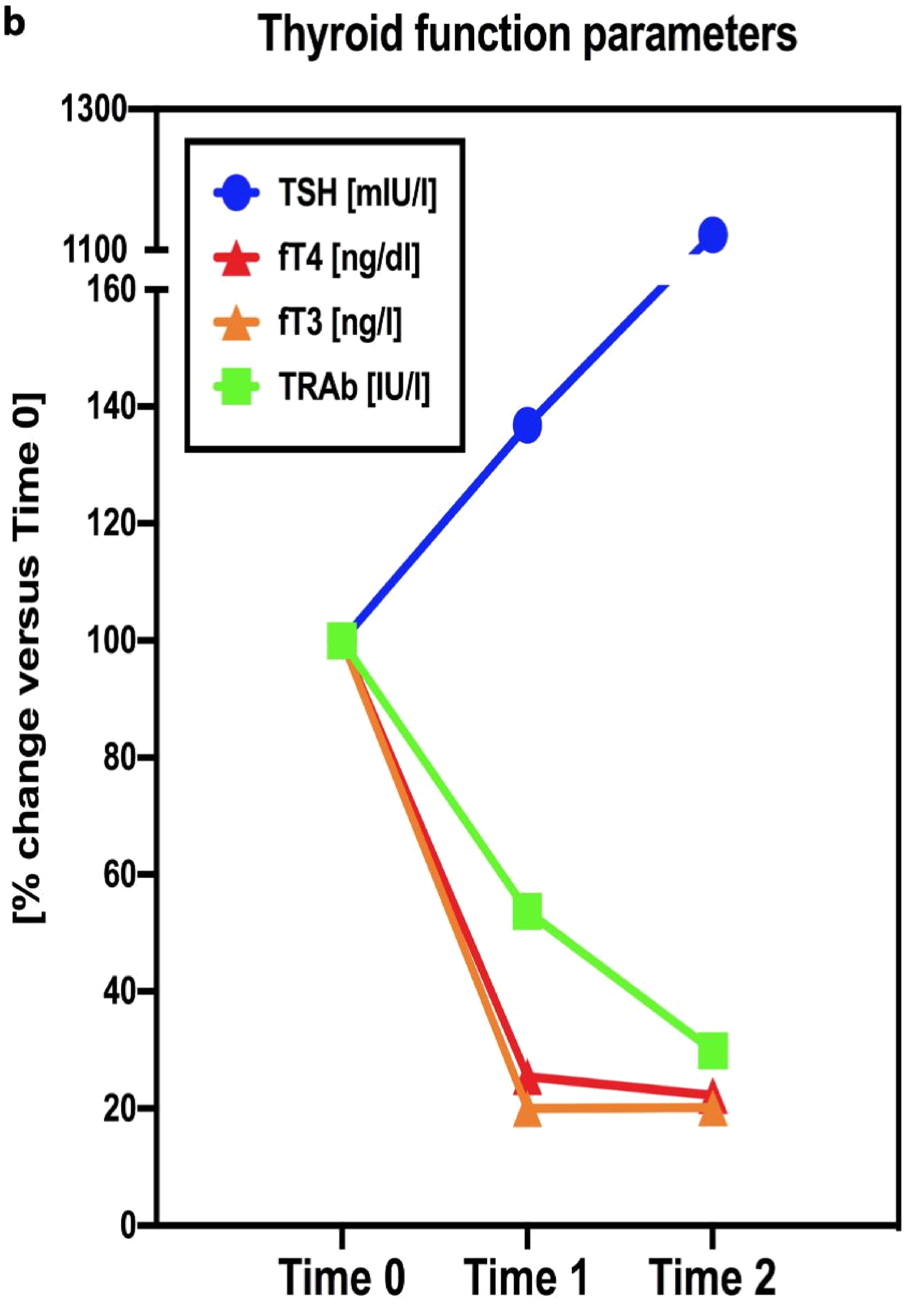

c

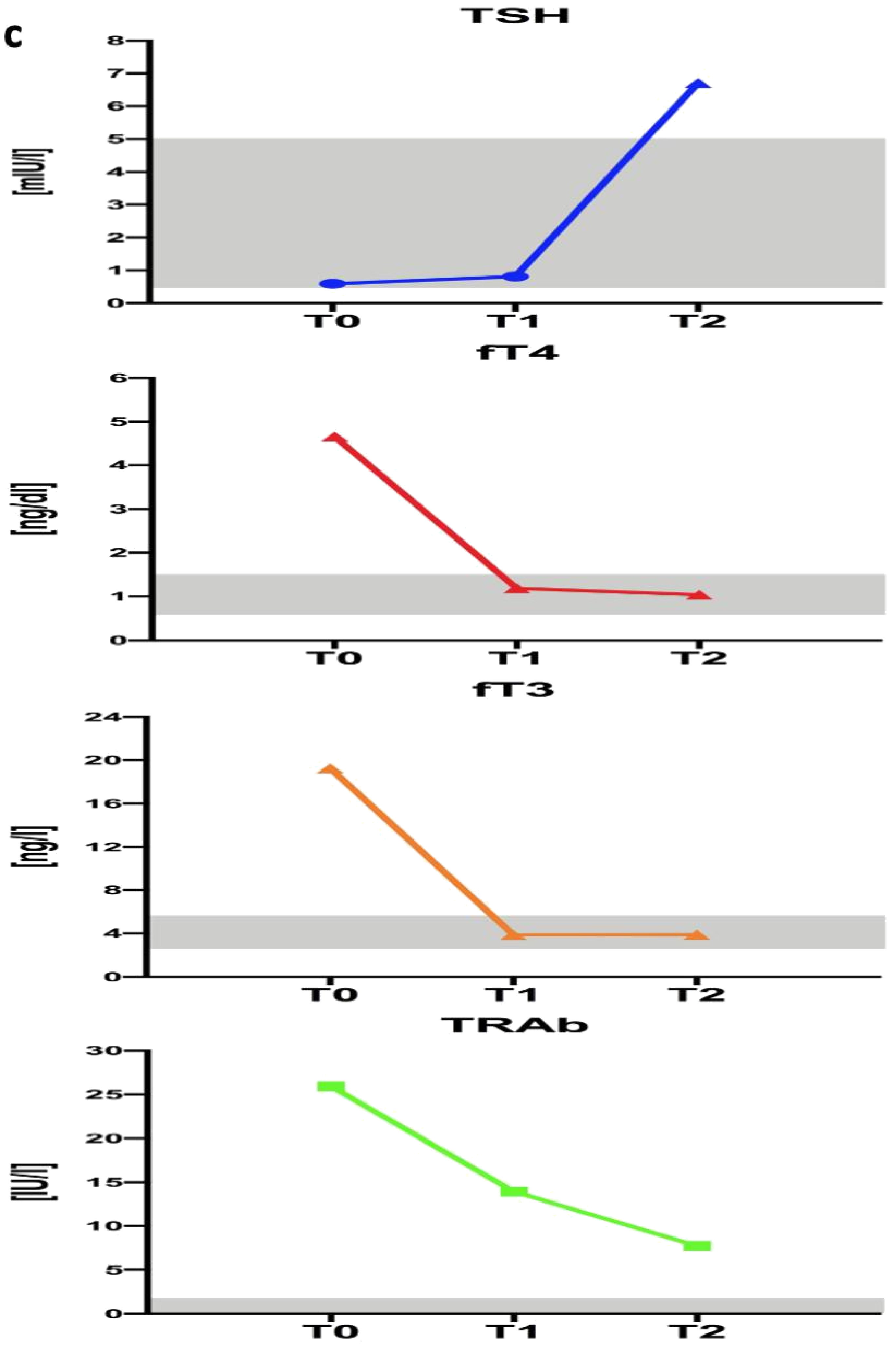

Supp. Tab. S1. Descriptive statistics of the studied groups. Tabular presentation of subjects characteristics and thyroid function-related parameters: TSH, free T4 (fT4), free T3 (fT3), TSH receptor antibodies (TRAb) (a). Treatment-related changes in thyroid parameters in the course of methimazole application: cumulative percentage change in laboratory tests results (b), and individual alterations in TSH, fT4, fT3 and TRAb (common units used; grey area indicate normal range values) (c). Data presented as mean values with standard deviation in the brackets.
